# The Greek version of the MacArthur competence assessment tool for treatment: reliability and validity. Evaluation of capacity for treatment decisions in Greek psychiatric patients

**DOI:** 10.1186/1744-859X-12-10

**Published:** 2013-04-09

**Authors:** Nikolaos Bilanakis, Aikaterini Vratsista, Georgios Kalampokis, Georgios Papamichael, Vaios Peritogiannis

**Affiliations:** 1 Department of Psychiatry, General Hospital of Arta, Arta, Greece; 2 Private Practice Psychiatrist, Ioannina, Greece

**Keywords:** Treatment decision-making capacity, MacArthur competence assessment tool for treatment, Competence, Psychiatric patients

## Abstract

**Background:**

Patients’ informed consent prior to treatment initiation is an essential component of contemporary clinical practice, but sometimes, patients lack decision-making capacity for treatment. Such capacity can be reliably assessed with standardized tools used, and the MacArthur competence assessment tool for treatment (MacCAT-T) is one of the most widely used instruments.

**Methods:**

The objective of this study was to translate the MacCAT-T into Greek and evaluate the Greek version’s reliability and validity in psychiatric patients. Thirty-nine psychiatric inpatients were examined with the MacCAT-T, and results showed an excellent inter-rater reliability.

**Results:**

Intraclass correlations ranged from 0.93 to 1 for the individual items of the tool. Severity of psychopathology was negatively correlated with reasoning, appreciation, and expressing a choice (Pearson’s *r* 0.36, 0.539, and 0.338, respectively), but there were no associations with demographic characteristics of the patients. Of the five factors derived from the brief psychiatric rating scale, anergia was significantly correlated with appreciation, reasoning, and expressing a choice (Pearson’s *r* 0.46, 0.45, and 0.37, respectively).

**Conclusions:**

The Greek version of the MacCAT-T is a reliable and valid instrument that can provide a standardized measure for assessing treatment decision capacity in Greek psychiatric patients and can be used for evaluation in the clinical practice.

## Introduction

In contemporary medical ethics, the right of the patient to accept or to refuse an offered treatment is recognized and respected. Patients’ informed consent prior to treatment initiation is an essential component of current clinical practice. Providing informed consent, however, requires patient’s decision-making capacity [1]. In certain cases, however, patients lack the capacity to make a decision about treatment. Hence, the determination of whether patients are capable is considered to be critical in striking a proper balance between respecting the autonomy of patients who are capable of making informed decisions and protecting those with cognitive impairment [2]. A systematic review of studies on psychiatric patients has shown that despite mental capacity being a complex and multidimensional construct, it can be reliably assessed when standardized assessments are used, as indicated by the high inter-rater reliability (median *k* = 0.81; IQR 0.75 to 0.82) when instruments were used to assist making a decision about capacity [3]. The authors also suggested that although incapacity is common, the majority of inpatients are capable of making treatment decisions, and this is in accordance with the results of a previous research [4]. However, in another study, rates of psychiatric inpatients’ incapacity were reported to be as high as 60% [5]. Similar results yielded a study in older psychiatric inpatients, 38.4% of whom had capacity for treatment decisions [6]. Another study, comparing 59 middle-aged and older patients diagnosed with schizophrenia or schizoaffective disorder and 38 normal comparison subjects, found that psychotic patients performed worse in the understanding item of the MacArthur competence assessment tool for treatment (MacCAT-T) [7]. Regarding patients diagnosed with schizophrenia, published studies demonstrate a substantial heterogeneity in decision-making capacity, and it has been suggested that the presence of schizophrenia does not necessarily mean the patient is incapable of making treatment decisions [8].

It has been suggested that an important part of the process of assessing patients’ capacities to make treatment decisions is the direct observation of their abilities to understand the meaning of information provided, appreciate its relevance for themselves, use the information in a decision-making process, and express a choice about treatment [9]. The MacCAT-T offers physicians and other health professionals practical guidance in their assessments of patients’ decision-making capacities in the context of informed consent to treatment [9]. The feasibility, reliability, and validity of this instrument have been tested in psychiatric patients, and previous studies have shown excellent inter-rater reliability (*k* > 0.8) for the MacCAT-T which offers a flexible yet structured method with which clinicians can assess, rate, and report patients’ abilities relevant for evaluating the capacity to consent to treatment [10]. The objective of the present study was to translate the MacCAT-T into Greek and evaluate the Greek version’s reliability and validity in psychiatric patients.

## Methods

### Patients

The patient sample was obtained from among all acutely ill patients admitted to the psychiatric ward of the General Hospital of Arta, north-western Greece over a 2-month period (November to December 2011). The institution’s ethics committee approved this study, and after full explanation of the study, signed informed consent was obtained from all participants. Every adult patient eligible for assessment in our 13-bed ward was screened for enrollment by a psychiatric consultant who served as the reference rater. Patients were excluded if they were non-Greek speaking, unwilling to participate, or too disorganized to be interviewed; if there was a chance of violent behavior toward the interviewer; and if the patient left the ward prior to being interviewed. The assessments and the clinical interviews were held during the first 72 h after admission. A total of 39 patients were recruited. The mean patient age was 44.8 years; most were men (59%), single, and had been admitted involuntarily. Two thirds of the patients had psychotic disorders, 28.2% of which were first-admitted and one third were multi-admitted patients (Table 1).

**Table 1:** **Patients**’ **characteristics**

**Variable**	**Sample (*n*= 39)**
Mean age (years)	44.8 (SD 12.057)
Men	23 (59%)
Single marital status	24 (61.5%)
Diagnosis	
Schizophrenia and related psychoses	26 (66.7%)
Bipolar disorder	7 (17.9%)
Depression	2 (5.1%)
Other	4 (10.3%)
Involuntary admission	23 (59%)
Number of admissions	
None	11 (28.2%)
1–2	10 (25.6%)
3–5	5 (12.8%)
>5	13 (33.3%)

### Measures

For the estimation of inter-rater reliability, each of the three consultant raters examined 13 different patients with the use of the MacCAT-T. All interviews were recorded, and the other two consultants rated independently the MacCAT-T for the other 26 patients based on recorded interviews. This means that every rater rated all the 39 patients: one third by clinical interview and two thirds by listening to the recorded interview of the patients with another rater. The MacCAT-T is a semi-structured interview, usually requiring about 15 to 20 min to complete that provides relevant information disclosures to patients about their illness, the nature of treatment options, and their risks and benefits. It guides the clinician through a disclosure of patients’ own disorders and treatment options. Questions to the patient require feedback, and this is used to assess the degree to which patients understand the information and recognize (appreciate) the relevance of the information for their own situation. Then, the clinician explores how patients are thinking through the treatment decision so as to estimate their reasoning abilities. Finally, the patient is asked to state a treatment choice, according to everything that has been considered [9]. The MacCAT-T has no cutoff score for incapacity, and this can be problematic when using its scores for statistical analysis. It may not be sufficient alone for the identification of the incapable patients but provides some evidence about treatment decision-making capacity. Judgment on capacity is guided by clinical assessment, including clinical interview and medical records review, and the rates on the MacCAT-T [4,11].

The MacCAT-T was translated from English into Greek by the first author (NB) and a group of three psychiatrists, using the back-translation method. This is a process of validity checking to make sure the translated version accurately reflects the item content of the original version (see Additional files 1 and 2) [12].

The 19-item version of the brief psychiatric rating scale (BPRS) was used to assess the severity of psychiatric symptoms.

### Statistical analysis

The MacCAT-T was completed for 39 inpatients by three raters (consultant psychiatrists) for each patient. Inter-rater reliability was estimated with the intraclass correlation coefficient index (ICC) for each of the four dimensions of the tool (understanding, appreciation, reasoning, and expressing a choice). Power analysis was conducted post hoc (see Additional file 3). All the statistical analyses were performed using the statistical package for the social sciences 19.0 (SPSS Inc, Chicago, IL, USA) for Windows and PASS 2008 v.08.0.11.

## Results

Intraclass correlations calculated among the three raters on the MacCAT-T summary ratings were 0.99 for understanding, 1 for appreciation, 0.98 for reasoning, and 0.96 for expressing a choice. Intraclass correlations ranged from 0.93 to 1 for the individual items of the MacCAT-T. The results show an excellent inter-rater reliability. The power for the observed values of inter-rater reliability, referring to a minimum of ICC of 0.9, exceeded 0.95, confirming the sample’s size adequacy for the conclusion reached.

For the estimation of the patients’ performance on the MacCAT-T, we used the values proposed in one of the original studies on the tool [10]. In understanding, a mean of 25.6% of the patients had ratings greater than 5, whereas, 52% had ratings of 4 or lower. In reasoning, there was a remarkable agreement between the three raters; ratings 3 or lower were found for 16 patients (41%). In appreciation, adequate summary ratings (3 or higher) were obtained by 53.8% of the patients. Clear deficiencies defined as ratings of 0 were found for only two subjects (5.1%) (Table 2).

**Table 2:** Ratings on scales of MacCAT-T by three raters and comparison with results of MacCAT-T study

**Scale and rating**	**Our study,*n*= 39 (%), mean of three raters**	**MacCAT-T study**[10]**,*n*= 40 (%)**
Understanding		
6 to 5.1	25.6	33
5 to 4.1	23	35
4 to 3.1	23	15
3 to 2.1	17.9	13
Less than 2.1	11	5
Reasoning		
8	7.7	20
7 to 6	27.3	33
5 to 4	23.9	18
3 to 2	18.7	5
1 to 0	21.3	15
Appreciation		
4	38.5	78
3	15.4	5
2	25.6	8
1	15.4	8
0	5.1	3

Regarding correlations of psychopathology, as measured with the BPRS and performance on the MacCAT-T, statistically significant results are presented in Table 3.

**Table 3:** **Correlations (Pearson’s *r*) between ratings on MacCAT-T scales and 19-item BPRS scores for 39 psychiatric inpatients**

**BPRS item**	**Understanding**	**Reasoning**	**Appreciation**	**Expressing a choice**
BPRS total score	0.038	0.360	0.539	0.338
Somatic concern				
Anxiety				
Withdrawal		0.51 (0.001)	0.42 (0.007)	0.36 (0.024)
Conceptual disorganization		0.34 (0.031)		0.36 (0.025)
Guilt feelings				
Tension				
Mannerisms		0.35 (0.028)		
Grandiosity				
Depressive mood				
Hostility				
Suspiciousness		0.41 (0.009)	0.57 (0.000)	0.33 (0.037)
Hallucinations				
Motor retardation				
Uncooperative			0.53 (0.000)	
Unusual thought			0.45 (0.004)	
Blunted affect		0.32 (0.049)	0.39 (.013)	
Excitement				
Disorientation	0.51 (0.001)		0.35 (0.029)	
Elevated mood				

*p* values are shown in brackets.

BPRS total scores were significantly related to MacCAT performance. More specifically, symptom severity was negatively correlated with reasoning, appreciation, and expressing a choice (Pearson’s *r* 0.36, 0.539, and 0.338, respectively). This is in accordance with previous research suggesting that illness severity is associated with decision-making capacity [3]. It also appears that several individual BPRS items were significantly correlated with MacCAT-T summary ratings. Disorientation was the only item negatively correlated to understanding, whereas withdrawal and suspiciousness were all correlated to reasoning, appreciation, and expressing a choice.

Regarding correlations of the five factors derived from BPRS, we have to note that anergia was significantly correlated with appreciation, reasoning, and expressing a choice (Pearson’s *r* 0.46, 0.45, and 0.37, respectively). This finding differs from earlier research [10] and should be interpreted with caution. However, it is interesting as it may suggest that negative symptoms strongly predict patient’s incapacity to make treatment decisions, while the same symptoms may make the patient susceptible to treatment decisions made by treating clinicians due to avolition and apathy. Negative symptoms are common and may predominate in the clinical picture in chronic psychotic patients. When these patients accept treatment, they may not in fact be capable for that decision. It is known that in several cases, patients may not accept the information about the disorder and the need for treatment, but they may still agree for the intervention [13]. Hostility and suspiciousness were also strongly associated with appreciation (*r* = 0.51) and reasoning (*r* = 0.33), in accordance with previous studies [10].

None of the ratings on MacCAT-T were significantly correlated with demographic characteristics of the patients, such as age, gender, education, and marital status nor was there a correlation or a trend for association between the number of prior admissions and performance on the MacCAT-T. Although the patient sample is small, this finding is in line with previous studies [4].

## Discussion

Assessment of patients’ decision-making capacity for treatment is significant in everyday clinical practice. Even more important is such assessment in psychiatric patients, especially inpatients, who may have been admitted involuntarily. Interestingly, a recent UK study suggested that most patients who regain capacity following psychiatric treatment indicated retrospective approval, even when initial treatment wishes had been overridden [11,14]. A wide variety of methods has been used by researchers to measure treatment decision-making capacity, and it is generally considered that it can be measured in a reliable manner [3]. Patients’ capacity can be reliably measured with the use of standardized instruments, and the MacCAT-T is one of the most widely used. Our study was intended to evaluate the Greek version of the MacCAT-T in psychiatric patients admitted to a general hospital psychiatric ward. This was the first effort to measure psychiatric patients’ capacity of making treatment decisions in Greece with the use of a standard assessment tool. In our country, patients’ capacity is determined merely by clinician’s judgment, based on the patient’s symptomatology and on other available information. In terms of validity, the results on the rating of MacCAT-T have demonstrated excellent inter-rater reliability in all the domains of the tool and suggest that different clinicians can rate MacCAT observations reliably.

We were able to directly compare our results only with the original study of the MacCAT-T [10]. As shown in Table 2, there were some significant differences in the instrument ratings. It should be mentioned that the MacCAT-T study involved 40 patients diagnosed with schizophrenia or schizoaffective disorder, whereas in our study, from a total of 39 patients, 66.7% had schizophrenia or related disorders and a further 17.9% were bipolar patients. Both diagnoses are related to lack of treatment decision-making capacity [5]. The most striking difference was in the item of appreciation, where 38.5% of our patients had a rating of 4, whereas in the MacCAT-T study, as many as 78% patients scored that much. Perhaps differences are better explained with the time of assessments, which were held during the first 72 h after admission in our study, but during the first 8 days in the MacCAT-T study (mean 4.2 days). It is possible that the intensive treatment during the first days of hospitalization would have improved patients’ acknowledgment of the disclosed information about the disorder (appreciation) and patients’ capacity to make treatment decisions. We believe that the validity of the Greek version of the MacCAT-T is further supported by this comparison, and this is in favor of the applicability of the tool for use within the Greek population.

It appears that a significant proportion of our patients lacked capacity for making treatment decisions, and this has been observed in several studies [4-6]. Several issues involving patient sample, study design, and measures may explain inconsistencies in reported frequencies of incapacity. As pointed out by Grisso and Appelbaum [9], capacity assessment not only involves psychopathology measurement, but also takes into account the patient’s functional abilities related to decision making, the current task demands, and the consequences of patient’s decisions. However, complete evaluation of patients’ treatment decision-making capacity with the consideration of clinical interview and medical records review, together with the scores on the MacCAT-T, was beyond the scope of our study.

Psychiatric patients are often not capable of making treatment decisions, and lack of capacity is associated with clinical and legal variables, such as psychotic disorders, illness severity, and involuntary admissions rather than with sociodemographic variables [3]. Severity of psychopathology, as measured by BPRS scores, was associated with incapacity in this study, in accordance with previous research [3]. Moreover, several BPRS items were correlated to individual domains of the MacCAT-T. These results should be interpreted with caution due to the small number of patients and the heterogeneity in patients’ diagnoses. These correlations might be completely different in larger and/or homogenous samples of patients. We did not analyze the correlation between the total BPRS scores and the total scores on the MacCAT-T as this approach has not be used so far by researchers, probably because it would be problematic to interpret the total score, if there was a low score in one single domain, suggesting incapacity.

Insight was not evaluated in this study. It has been suggested, however, that low insight is associated with incapacity and that among all the clinical constructs, insight is the strongest discriminator of capacity status [11]. A recent systematic review of the literature included seven studies of psychiatric inpatients and outpatients, all of which had used the MacCAT-T for capacity estimation, and all studies but one found a strong correlation between poor insight and incapacity. This was particularly the case for psychotic patients with poor insight [15].

The study has some limitations. The number of patients is small, and conclusions regarding association of certain aspects of psychopathology with capacity to consent to treatment cannot be drawn. Another limitation is that the study involves only inpatients. Psychiatric inpatients may temporarily have limited decisional capacity for a variety of reasons, including greater symptom severity, experiencing hospitalization as a stressful life event, and receiving high doses of psychotropic medications which may reduce cognitive performance [8]. Perhaps, psychiatric outpatients would perform better in the MacCAT-T. Regarding patients who were excluded from our study, we assume that disorganized or violent patients, as well as those who left the ward against clinician advice, would not perform well to the MacCAT-T within the assessment period of 72 h.

## Conclusions

In conclusion, we believe that results are satisfactory and show that the Greek version of the MacCAT-T is a practical, reliable, and valid instrument that can provide a standardized measure for assessing treatment decision capacity in Greek psychiatric patients and can be used for evaluation in clinical practice. Further study on outpatients and inpatients with the use of MacCAT will clarify whether certain aspects of psychopathology may help clinicians in predicting incapacity for treatment decisions in such patients.

## Competing interests

The authors declare that they have no competing interests.

## Authors’ contributions

NB conceived the study, participated in its design and coordination, and helped draft the manuscript. AV, GK, and GP participated in the study design and collected the data. VP helped in the data collection and the writing of the manuscript. All authors read and approved the final manuscript.

## Supplementary Material

Additional file 1 The Greek version of the MacCAT. Click here for file

Additional file 2 Translation examples of the Greek version of the MacCAT. Click here for file

Additional file 3 **Power analysis graph.** The power for the observed values of inter-rater reliability exceeds 0.95 and confirms the sample’s size adequacy. Click here for file
