# Supplementary material for: The Greek version of the MacArthur competence assessment tool for treatment: reliability and validity. Evaluation of capacity for treatment decisions in Greek psychiatric patients
Source: Ann Gen Psychiatry. 2013 Apr 9;12:10. doi: 10.1186/1744-859X-12-10 (PMC3648422; doi:10.1186/1744-859X-12-10)
Supplement: Additional file 1 — The Greek version of the MacCAT. [file 1744-859X-12-10-S1.doc]

**MacCAT-T Φύλο Καταγραφής**

(Μετάφραση-Προσαρμογή στα ελληνικά: Ν. Μπιλανάκης)

Ονοματεπώνυμο ασθενούς:……………………………………………………………

Ονοματεπώνυμο κλινικού:……………………………………………………………..

# Ημερομηνία:…………………………..Ώρα:…………Μονάδα:………………………

## ΚΑΤΑΝΟΗΣΗ (ΤΗΣ ΔΙΑΤΑΡΑΧΗΣ)

Πληροφόρησε τον/την ασθενή για την ασθένεια του/της. Εξέτασε: «Σας παρακαλώ να μου πείτε με δικά σας λόγια τι σας είπα για την ασθένεια σας». (Αν δεν απαντά ή απαντά λάθος): Ξαναπληροφόρησε και επανεξέτασε.

**Πληροφορίες προς παροχή** **Απαντήσεις ασθενούς**

| 1. Διάγνωση της Διαταραχής (όνομα)  ………………………………………….. |  | Βαθμός |
| --- | --- | --- |
|  |  |  |
| 2. Χαρακτηριστικά της Διαταραχής (επέλεξε 1 από τα 3 πιο σημαντικά χαρ/κά της ασθένειας, όπως π.χ. βιολογικούς μηχανισμούς, αιτίες, σημεία, που θεωρείς αναγκαία να κατανοήσει ο ασθενής ώστε να αποφασίσει)  ………………………………………….  ………………………………………….  …………………………………………. |  |  |
|  |  | Βαθμός |
|  |  |  |
| 3. Χαρακτηριστικά της Διαταραχής (επέλεξε 1 από τα 3 πιο σημαντικά χαρ/κά της ασθένειας, όπως π.χ. βιολογικούς μηχανισμούς, αιτίες, σημεία, που θεωρείς αναγκαία να κατανοήσει ο ασθενής ώστε να αποφασίσει)  …………………………………………...  …………………………………………... |  |  |
|  |  | Βαθμός |
|  |  |  |
| 4. Χαρακτηριστικά της Διαταραχής (επέλεξε 1 από τα 3 πιο σημαντικά χαρ/κά της ασθένειας, όπως π.χ. βιολογικούς μηχανισμούς, αιτίες, σημεία, που θεωρείς αναγκαία να κατανοήσει ο ασθενής ώστε να αποφασίσει)  …………………………………………...  …………………………………………… |  |  |
|  |  | Βαθμός |
|  |  |  |
| 5. Πορεία της Διαταραχής (Περιέγραψε τις συνέπειες που θα προκύψουν εφόσον δεν δοθεί θεραπεία στη Διαταραχή).  ……………………………………………………………………………………………………………………………………………………………… |  | Βαθμός |
|  |  |  |

### ΚΑΤΑΝΟΗΣΗ (ΤΗΣ ΔΙΑΤΑΡΑΧΗΣ) (Συνολικός Βαθμός)

| Άλλο |  |
| --- | --- |

**ΑΝΑΓΝΩΡΙΣΗ (ΤΗΣ ΔΙΑΤΑΡΑΧΗΣ)**

(Προσδιόρισε αν α) ο/η ασθενής αναγνωρίζει ότι αυτός/αυτή έχει την διαταραχή και τα συμπτώματα που του/της περιγράφηκαν ή αν β) οι εναλλακτικές εξηγήσεις που δίνει ο/η ασθενής βασίζονται σε λογικά δεδομένα (π.χ. για τα ίδια συμπτώματα είχε δεχθεί παλαιότερα άλλη διάγνωση ή σε θρησκευτικές και πολιτισμικές πεποιθήσεις.)

Ήδη γνωρίζεις ποιο, σύμφωνα με τη γνώμη των γιατρών, είναι το πρόβλημα στη δική σου περίπτωση. Αν για κάποιο λόγο δεν συμφωνείς με αυτή τη γνώμη, θάθελα να μου το πεις. Λοιπόν, τι νομίζεις;

Συμφωνεί Διαφωνεί Αμφιβάλλει

(Αν ο ασθενής διαφωνεί ή αμφιβάλλει, ο κλινικός μέσω των εξηγήσεων του ασθενούς οφείλει να προσδιορίσει τη βάση αυτής της διαφωνίας και εάν είναι ακλόνητη ή μπορεί εύκολα να αλλάξει.)

| Εξήγηση ασθενούς  ………………………………………………………………………………..  ……………………………………………………………………………….. | Βαθμός |
| --- | --- |
|  |

**ΚΑΤΑΝΟΗΣΗ (ΤΗΣ ΘΕΡΑΠΕΙΑΣ)**

Πληροφόρησε τον/την ασθενή για την συνιστώμενη θεραπεία. Εξέτασε: «Σας παρακαλώ να μου πείτε με δικά σας λόγια τι σας είπα για την θεραπεία». (Αν δεν απαντά ή απαντά λάθος): Ξαναπληροφόρησε και επανεξέτασε.

**Πληροφορίες προς παροχή** **Απαντήσεις ασθενούς**

| 1. Όνομα της Θεραπείας  ………………………………………….. |  | Βαθμός |
| --- | --- | --- |
|  |  |  |
| 2. Χαρακτηριστικά της Θεραπείας (επέλεξε 1 από τα 3 πιο σημαντικά χαρ/κά της θεραπείας που θεωρείς αναγκαία να κατανοήσει ο ασθενής, όπως π.χ. μεθοδολογία χορήγησης αγωγής, απαιτούμενος χρόνος λήψης της, ώστε να αποφασίσει για την θεραπεία του)  ……………………………………………………………………………………….. |  |  |
|  |  | Βαθμός |
|  |  |  |
| 3. Χαρακτηριστικά της Διαταραχής (επέλεξε 1 από τα 3 πιο σημαντικά χαρ/κά της ασθένειας που θεωρείς αναγκαία να κατανοήσει ο ασθενής, όπως π.χ. μεθοδολογία χορήγησης αγωγής, απαιτούμενος χρόνος λήψης της, ώστε να αποφασίσει για την θεραπεία του)  …………………………………………...  …………………………………………... |  |  |
|  |  | Βαθμός |
|  |  |  |
| 4. Χαρακτηριστικά της Διαταραχής (επέλεξε 1 από τα 3 πιο σημαντικά χαρ/κά της ασθένειας που θεωρείς αναγκαία να κατανοήσει ο ασθενής, όπως π.χ. μεθοδολογία χορήγησης αγωγής, απαιτούμενος χρόνος λήψης της, ώστε να αποφασίσει για την θεραπεία του)  …………………………………………...  …………………………………………… |  |  |
|  |  | Βαθμός |
|  |  |  |

**ΚΑΤΑΝΟΗΣΗ (ΤΗΣ ΘΕΡΑΠΕΙΑΣ)** (Συνολικός Βαθμός)

**ΚΑΤΑΝΟΗΣΗ (ΩΦΕΛΕΙΩΝ-ΚΙΝΔΥΝΩΝ ΑΠΟ ΤΗΝ ΘΕΡΑΠΕΙΑ)**

Πληροφόρησε τον/την ασθενή για τα οφέλη και τους κινδύνους που αναμένονται από τη θεραπεία. Εξέτασε: «Σας παρακαλώ να μου πείτε με δικά σας λόγια τι σας είπα για τις ωφέλειες και κινδύνους από αυτή τη θεραπεία. (Αν δεν απαντά ή απαντά λάθος): Ξαναπληροφόρησε και επανεξέτασε.

Πληροφορίες προς παροχή Απαντήσεις ασθενούς

| 1. Οφέλη (Προσδιόρισε 1 από 2 πιο σημαντικά αναμενόμενα οφέλη από την θεραπεία καθώς και την πιθανότητα να συμβεί)  ………………………………………….. |  | Βαθμός |
| --- | --- | --- |
|  |  |  |
| 2. Οφέλη (Προσδιόρισε 1 από 2 πιο σημαντικά αναμενόμενα οφέλη από την θεραπεία καθώς και την πιθανότητα να συμβεί)  ……………………………………………………………………………………….. |  |  |
|  |  | Βαθμός |
|  |  |  |
| 3. Κίνδυνοι (Προσδιόρισε 1 από 2 πιο σημαντικούς κίνδυνους, ενοχλήσεις ή παρενέργειες από την θεραπεία καθώς και την πιθανότητα να συμβεί)  …………………………………………...  …………………………………………... |  |  |
|  |  | Βαθμός |
|  |  |  |
| 4. Κίνδυνοι (Προσδιόρισε 1 από 2 πιο σημαντικούς κίνδυνους, ενοχλήσεις ή παρενέργειες από την θεραπεία καθώς και την πιθανότητα να συμβεί)  …………………………………………...  …………………………………………… |  |  |
|  |  | Βαθμός |
|  |  |  |

**Κατανόηση Ωφελειών-Κινδύνων από την θεραπεία** (Συνολικός βαθμός)

| Άλλο |  |
| --- | --- |

**ΑΝΑΓΝΩΡΙΣΗ (ΤΗΣ ΑΞΙΑΣ ΤΗΣ ΘΕΡΑΠΕΙΑΣ)**

Δεν αποτελεί σκοπό αυτής της ερώτησης ο προδιορισμός του αν ο ασθενής αποδέχεται την προτεινόμενη θεραπεία. Προσδιόρισε: α) αν ο ασθενής αναγνωρίζει ότι η προτεινόμενη θεραπεία θα μπορούσε να αποβεί σε όφελος του και, αν όχι, β) προσδιόρισε αν τα επιχειρήματα που προβάλλει ο ασθενής για να υποστηρίξει την αντίθετη γνώμη βασίζονται σε παλαιότερες εμπειρίες που λογικά οδηγούν σε αυτό το συμπέρασμα (π.χ. ο ασθενής έλαβε στο παρελθόν την προτεινόμενη θεραπεία χωρίς σημαντικό όφελος) ή σε θρησκευτικές ή πολιτιστικές δοξασίες. Προσδιόρισε επίσης αν η αναγνώριση ή η μη αναγνώριση της πιθανής αξίας της θεραπείας οφείλεται σε συγχυτικές, παραληρητικές ή συναισθηματικές καταστάσεις.

Εξέτασε: «Μπορεί να αποφάσισες ή να μην αποφάσισες ότι αυτή είναι η θεραπεία που θέλεις- θα μιλήσουμε γι’αυτό αργότερα. Πιστεύεις όμως ότι είναι πιθανό αυτή η θεραπεία να μπορούσε να αποβεί σε όφελος σου;».

Συμφωνεί Διαφωνεί Αμφιβάλλει

Επέμεινε. «Νοιώθεις ότι είναι/δεν είναι πιθανό αυτή η θεραπεία να αποβεί σε όφελος σου. Μπορείς να μου το εξηγήσεις; Τι σε κάνει να πιστεύεις ότι αυτή η θεραπεία είναι/δεν είναι πιθανό να αποβεί σε όφελος σου;

| Εξήγηση ασθενούς  ………………………………………………………………………………..  ……………………………………………………………………………….. | Βαθμός |
| --- | --- |

**ΕΝΑΛΛΑΚΤΙΚΕΣ ΘΕΡΑΠΕΙΕΣ**

Χρησιμοποίησε τα Φύλα Καταγραφής των Εναλλακτικών Θεραπειών, ένα Φύλο για κάθε Θεραπεία.

Η συμπλήρωση αυτού του μέρους του Φύλου Καταγραφής δεν είναι απαραίτητη για την αξιολόγηση της ικανότητας του ασθενούς να αποφασίζει. Αυτό το μέρος μπορεί να φανεί χρήσιμο σε περιπτώσεις στις οποίες η πιστοποίηση της κατανόησης όλων των διαθέσιμων θεραπειών είναι επιθυμητή, όπως συμβαίνει σε δικαστικές περιπτώσεις.

**ΠΡΩΤΗ ΘΕΡΑΠΕΥΤΙΚΗ ΕΠΙΛΟΓΗ ΚΑΙ ΓΝΩΣΙΑΚΗ ΑΚΟΛΟΥΘΙΑ**

**Ρώτησε: «**Ας ανασκοπήσουμε τις θεραπευτικές επιλογές που έχεις. Πρώτη...... Δεύτερη..... κ.ο.κ. (ανέφερε κάθε θεραπευτική επιλογή που έχει ήδη αναφερθεί, συμπεριλαμβανομένης και της μη παροχής θεραπείας.) Ποιά από αυτές φαίνεται καλύτερη για σένα; Ποιά πιστεύεις ότι είναι πιθανότερο να θέλεις;»

**Πρώτη Επιλογή**:.............................................................................................................

..........................................................................................................................................

..........................................................................................................................................

(κατέγραψε όλες τις επιλογές αν είναι περισσότερες από μία).

**Ρώτησε:** «Πιστεύεις ότι η ....(ανέφερε την επιλογή του ασθενούς) μπορεί να είναι η καλύτερη επιλογή. Πές μου τι την κάνει να φαίνεται καλύτερη από τις άλλες;»

Συζήτησε τις εξηγήσεις του ασθενούς ώστε να διερευνήσεις τη γνωσιακή ακολουθία.

| Εξήγηση ασθενούς  ………………………………………………………………………………..  ………………………………………………………………………………..  ............................................................................................................................................................................................................................................................................................................................................................................................................................................................................................................ | | Επαγωγική σκέψη | | --- | |  | | Συγκριτική σκέψη | |  | |
| --- | --- | --- | --- | --- | --- |

**ΣΥΝΕΠΕΙΕΣ**

Στόχος αυτού του τμήματος είναι ο προσδιορισμός της ικανότητας του ασθενούς να μεταφράσει τις ιατρικές παραμέτρους της διαταραχής και της θεραπείας (π.χ. συμπτώματα, ωφέλη και κίνδυνοι από τη θεραπεία) σε πρακτικές συνέπειες της καθημερινότητας ( π.χ. επίπτωση στις διαπροσωπικές σχέσεις, στην εργασία κλπ).

**Ερώτηση 1:** «Σας είπα για κάποια από τα πιθανά ωφέλη και κινδύνους ή ενοχλήσεις από ...(ανάφερε την προτειμόμενη από τον ασθενή θεραπεία). Πείτε μου κάποιους τρόπους με τους οποίους αυτά μπορούν να επιρεάσουν τις καθημερινές δραστηριότητες σας στο σπίτι ή στην εργασία».

| Συνέπειες 1. | Συνέπειες 1 |
| --- | --- |
|  |

**Ερώτηση 2:** «Τώρα, ας δούμε τις ...(ανάφερε οποιαδήποτε άλλη εναλλακτική θεραπεία ή την μη παροχή θεραπείας). Πείτε μου κάποιους τρόπους με τους οποίους η έκβαση όλων αυτών μπορεί να επιρεάσει τις καθημερινές δραστηριότητες σας στο σπίτι ή στην εργασία».

| Συνέπειες 2. | Συνέπειες 2 |
| --- | --- |
|  |

**ΣΥΝΕΠΕΙΕΣ** **(τελικός βαθμός):**

**ΤΕΛΙΚΗ ΕΠΙΛΟΓΗ**

**Ερώτηση: «**Όταν ξεκινήσαμε αυτή τη συζήτηση προτιμήσατε ...(ανέφερε την *Πρώτη Επιλογή* από προηγούμενο τμήμα ή σημείωσε ότι ο ασθενής μοιάζει να έχει δυσκολίες να αποφασίσει). Τι πιστεύετε τώρα που έχουμε συζητήσει όλες τις επιλογές; Ποιά επιθυμείτε να ακολουθήσετε;»

| Επιλογή. | Έκφραση Επιλογής |
| --- | --- |
|  |

Εκτίμησε το κατά πόσο η τελική επιλογή προκύπτει λογικά από την προηγούμενη γνωσιακή ακολουθία και τις συνέπειες. Αν ναί, δεν χρειάζεται να προχωρήσετε. Αν όχι, συζήτησε τη μη συνάφεια με τον ασθενή και κατέγραψε τη διαδικασία παρακάτω.

**ΛΟΓΙΚΗ ΣΥΝΑΦΕΙΑ ΤΗΣ ΕΠΙΛΟΓΗΣ**

| Εξήγηση εξεταστού. | Λογική συνάφεια |
| --- | --- |
|  |

**Περιληπτική Βαθμολόγηση Ερωτηματολογίου.**

**ΚΑΤΑΝΟΗΣΗ** Συνολική Αριθμός Υφολική

Βαθμολογία / Ερωτήσεων Βαθμολογία

| Διαταραχής | **………………** | **…………… =** | **……………………** |
| --- | --- | --- | --- |
| Θεραπείας | **………………** | **…………… =** | **……………………** |
| Οφέλη / Κίνδυνοι | **………………** | **…………… =** | **……………………** |

**Κατανόηση Περιληπτική Βαθμολογία (0-6)**

ΑΝΑΓΝΩΡΙΣΗ

| Διαταραχής ...……………… |  |
| --- | --- |
| Θεραπείας ………………… |  |

**Αναγνώριση Περιληπτική Βαθμολογία (0-4)**

ΓΝΩΣΙΑΚΗ ΑΚΟΛΟΥΘΙΑ

| Επαγωγική ………………… |  |
| --- | --- |
| Συγκριτική ….. ……………. |  |
| Συνέπειες ………………… |  |
| Λογική συνάφεια ………………… |  |

**Γνωσιακή ακολουθία Περιληπτική Βαθμολογία (0-8)**

**Έκφραση επιλογής Περιληπτική Βαθμολογία (0-2)**

**ΠΡΟΑΙΡΕΤΙΚΟ: Περιληπτική βαθμολογία κάθε μίας εναλλακτικής θεραπείας**

Εναλλακτική Θεραπεία 1:

Εναλλακτική Θεραπεία 2:

Εναλλακτική Θεραπεία 3:

Εναλλακτική Θεραπεία 4:

**ΦΥΛΟ ΚΑΤΑΓΡΑΦΗΣ ΕΝΑΛΛΑΚΤΙΚΩΝ ΘΕΡΑΠΕΙΩΝ**

**Ασθενής:………………………………………………………………………………**

**ΚΑΤΑΝΟΗΣΗ (ΤΗΣ ΘΕΡΑΠΕΙΑΣ)**

Πληροφόρησε τον/την ασθενή για κάθε μία εναλλακτική θεραπεία ξεχωριστά. Εξέτασε: «Σας παρακαλώ να μου πείτε με δικά σας λόγια τι σας είπα για την θεραπεία». (Αν δεν απαντά ή απαντά λάθος): Ξαναπληροφόρησε και επανεξέτασε.

**Πληροφορίες προς παροχή** **Απαντήσεις ασθενούς**

| 1. Όνομα της Θεραπείας  ………………………………………….. |  | Βαθμός |
| --- | --- | --- |
|  |  |  |
| 2. Χαρακτηριστικά της Θεραπείας (επέλεξε 1 από τα 3 πιο σημαντικά χαρ/κά της θεραπείας που θεωρείς αναγκαία να κατανοήσει ο ασθενής, όπως π.χ. μεθοδολογία χορήγησης αγωγής, απαιτούμενος χρόνος λήψης της, ώστε να αποφασίσει για την θεραπεία του)  ……………………………………………………………………………………….. |  |  |
|  |  | Βαθμός |
|  |  |  |
| 3. Χαρακτηριστικά της Διαταραχής (επέλεξε 1 από τα 3 πιο σημαντικά χαρ/κά της ασθένειας που θεωρείς αναγκαία να κατανοήσει ο ασθενής, όπως π.χ. μεθοδολογία χορήγησης αγωγής, απαιτούμενος χρόνος λήψης της, ώστε να αποφασίσει για την θεραπεία του)  …………………………………………...  …………………………………………... |  |  |
|  |  | Βαθμός |
|  |  |  |
| 4. Χαρακτηριστικά της Διαταραχής (επέλεξε 1 από τα 3 πιο σημαντικά χαρ/κά της ασθένειας που θεωρείς αναγκαία να κατανοήσει ο ασθενής, όπως π.χ. μεθοδολογία χορήγησης αγωγής, απαιτούμενος χρόνος λήψης της, ώστε να αποφασίσει για την θεραπεία του)  …………………………………………...  …………………………………………… |  |  |
|  |  | Βαθμός |
|  |  |  |

**ΚΑΤΑΝΟΗΣΗ (ΤΗΣ ΘΕΡΑΠΕΙΑΣ)** (Συνολικός Βαθμός)

| Άλλο |  |
| --- | --- |

**ΚΑΤΑΝΟΗΣΗ (ΩΦΕΛΕΙΩΝ-ΚΙΝΔΥΝΩΝ ΑΠΟ ΤΗΝ ΘΕΡΑΠΕΙΑ)**

Πληροφόρησε τον/την ασθενή για τα οφέλη και τους κινδύνους που αναμένονται από τη θεραπεία. Εξέτασε: «Σας παρακαλώ να μου πείτε με δικά σας λόγια τι σας είπα για τις ωφέλειες και κινδύνους από αυτή τη θεραπεία. (Αν δεν απαντά ή απαντά λάθος): Ξαναπληροφόρησε και επανεξέτασε.

Πληροφορίες προς παροχή Απαντήσεις ασθενούς

| 1. Οφέλη (Προσδιόρισε 1 από 2 πιο σημαντικά αναμενόμενα οφέλη από την θεραπεία καθώς και την πιθανότητα να συμβεί)  ………………………………………….. |  | Βαθμός |
| --- | --- | --- |
|  |  |  |
| 2. Οφέλη (Προσδιόρισε 1 από 2 πιο σημαντικά αναμενόμενα οφέλη από την θεραπεία καθώς και την πιθανότητα να συμβεί)  ……………………………………………………………………………………….. |  |  |
|  |  | Βαθμός |
|  |  |  |
| 3. Κίνδυνοι (Προσδιόρισε 1 από 2 πιο σημαντικούς κίνδυνους, ενοχλήσεις ή παρενέργειες από την θεραπεία καθώς και την πιθανότητα να συμβεί)  …………………………………………...  …………………………………………... |  |  |
|  |  | Βαθμός |
|  |  |  |
| 4. Κίνδυνοι (Προσδιόρισε 1 από 2 πιο σημαντικούς κίνδυνους, ενοχλήσεις ή παρενέργειες από την θεραπεία καθώς και την πιθανότητα να συμβεί)  …………………………………………...  …………………………………………… |  |  |
|  |  | Βαθμός |
|  |  |  |

**Κατανόηση Ωφελειών-Κινδύνων από την θεραπεία** (Συνολικός βαθμός)

| Άλλο |  |
| --- | --- |
