# Supplementary material for: The Greek version of the MacArthur competence assessment tool for treatment: reliability and validity. Evaluation of capacity for treatment decisions in Greek psychiatric patients
Source: Ann Gen Psychiatry. 2013 Apr 9;12:10. doi: 10.1186/1744-859X-12-10 (PMC3648422; doi:10.1186/1744-859X-12-10)
Supplement: Additional file 2 — Translation examples of the Greek version of the MacCAT. [file 1744-859X-12-10-S2.docx]

**Translation examples**

APPRECIATION-DISORDER

“Now that is what we think is the problem in your case. If you have any reason to doubt that, I’d like you to tell me so. What do you think?”

**In Greek:** Ήδη γνωρίζεις ποιο, σύμφωνα με τη γνώμη των γιατρών, είναι το πρόβλημα στη δική σου περίπτωση. Αν για κάποιο λόγο δε συμφωνείς με αυτή τη γνώμη, θά’θελα να μου το πεις. Λοιπόν, τι νομίζεις;

UNDERSTANDING-BENEFITS/RISKS

“Now please explain in your own words what I’ve said about benefits and risks of this treatment”

**In Greek:** Σας παρακαλώ να μου πείτε με δικά σας λόγια τι σας είπα για τις ωφέλειες και κινδύνους από αυτή τη θεραπεία

APPRECIATION-TREATMENT

“You might or might not decide that this is the treatment you want-we’ll talk about it later. But do you think it’s possible that this treatment might be of some benefit to you?”

**In Greek:** Μπορεί να αποφάσισες ή να μην αποφάσισες ότι αυτή είναι η θεραπεία που θέλεις- θα μιλήσουμε γι’αυτό αργότερα. Πιστεύεις όμως ότι είναι πιθανό αυτή η θεραπεία να μπορούσε να αποβεί σε όφελος σου;

GENERATE CONSEQUENCES

Inquire-1: “I told you about some of the possible benefits and risks or discomforts of (name the patient’s preferred treatment option). What are some ways that these might influence your everyday activities at home or at work?”

**In Greek:** Σας είπα για κάποια από τα πιθανά ωφέλη και κινδύνους ή ενοχλήσεις από ...(ανάφερε την προτιμώμενη από τον ασθενή θεραπεία). Πείτε μου κάποιους τρόπους με τους οποίους αυτά μπορούν να επηρεάσουν τις καθημερινές δραστηριότητες σας στο σπίτι ή στην εργασία

FINAL CHOICE

“When we started this discussion you favored (insert “First Choice” from earlier inquiry, or note that the patient seemed to be having difficulty deciding). What do you think now that we have discussed everything? Which do you want to do?”

In Greek: Όταν ξεκινήσαμε αυτή τη συζήτηση προτιμήσατε ...(ανέφερε την Πρώτη Επιλογή από προηγούμενο τμήμα ή σημείωσε ότι ο ασθενής μοιάζει να έχει δυσκολίες να αποφασίσει). Τι πιστεύετε τώρα που έχουμε συζητήσει όλες τις επιλογές; Ποιά επιθυμείτε να ακολουθήσετε;

**Comment**

**It generally appears that questions in Greek are simple and understandable for patients, even low-education patients. Patients with schizophrenia also understood these questions well.**
