# Supplementary figures and images for: The Greek version of the MacArthur competence assessment tool for treatment: reliability and validity. Evaluation of capacity for treatment decisions in Greek psychiatric patients
Source: Ann Gen Psychiatry. 2013 Apr 9;12:10. doi: 10.1186/1744-859X-12-10 (PMC3648422; doi:10.1186/1744-859X-12-10)

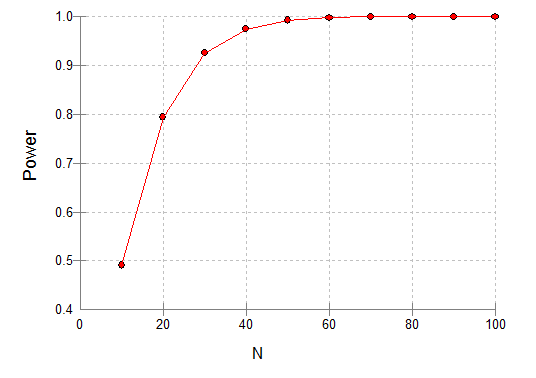

Supplement: Additional file 3 — Power analysis graph. The power for the observed values of inter-rater reliability exceeds 0.95 and confirms the sample’s size adequacy. [file 1744-859X-12-10-S3.docx]
